# Maxillary Overdentures Supported by Four Splinted Direct Metal Laser Sintering Implants: A 3-Year Prospective Clinical Study

**DOI:** 10.1155/2014/252343

**Published:** 2014-12-14

**Authors:** Francesco Mangano, Fabrizia Luongo, Jamil Awad Shibli, Sukumaran Anil, Carlo Mangano

**Affiliations:** ^1^Department of Surgical and Morphological Science, Dental School, University of Varese, Via Giuseppe Piatti 10, 21100 Varese, Italy; ^2^Private Practice, 00193 Rome, Italy; ^3^Dental Research Division, Department of Periodontology, Guarulhos University, Praca Teresa Cristina 229, 07023070 Guarulhos, SP, Brazil; ^4^Division of Periodontics, College of Dentistry, King Saud University, P.O. Box 60169, Riyadh 11545, Saudi Arabia

## Abstract

*Purpose*. Nowadays, the advancements in direct metal laser sintering (DMLS) technology allow the fabrication of titanium dental implants. The aim of this study was to evaluate implant survival, complications, and peri-implant marginal bone loss of DMLS implants used to support bar-retained maxillary overdentures.* Materials and Methods*. Over a 2-year period, 120 implants were placed in the maxilla of 30 patients (18 males, 12 females) to support bar-retained maxillary overdentures (ODs). Each OD was supported by 4 implants splinted by a rigid cobalt-chrome bar. At each annual follow-up session, clinical and radiographic parameters were assessed. The outcome measures were implant failure, biological and prosthetic complications, and peri-implant marginal bone loss (distance between the implant shoulder and the first visible bone-to-implant contact, DIB).* Results*. The 3-year implant survival rate was 97.4% (implant-based) and 92.9% (patient-based). Three implants failed. The incidence of biological complication was 3.5% (implant-based) and 7.1% (patient-based). The incidence of prosthetic complication was 17.8% (patient-based). No detrimental effects on marginal bone level were evidenced.* Conclusions*. The use of 4 DMLS titanium implants to support bar-retained maxillary ODs seems to represent a safe and successful procedure. Long-term clinical studies on a larger sample of patients are needed to confirm these results.

## 1. Introduction

For many years, removable complete dentures have represented the main solution for restoration of fully edentulous patients [1–3]. Although many edentulous patients are satisfied with their complete dentures, some encounter problems with this design, including bone resorption of residual ridges over time and lack of stability and retention, with decreased chewing ability [1–3]. This decreased masticatory efficiency can affect overall nutritional intake, comfort, and self-confidence; these limitations often cause social and psychological disabilities, and are associated with reduced quality of life [1–3]. Nowadays, these problems can be successfully addressed with the placement of endosseous dental implants in the edentulous jaws, to retain overdentures (ODs) [3]. The benefits of an implant-supported OD include increased stability and retention, improvement in chewing ability, and comfort, resulting in higher patient satisfaction and oral health-related quality of life [1–3]. Although several studies and systematic reviews have dealt with implant-supported mandibular ODs, reporting excellent long-term outcomes [4–6], only a few studies reported on mid- and long-term results with implant-supported maxillary ODs [7–10]; in addition, less favorable mid-term and long-term implant survival/success rates have been reported for maxillary implants supporting ODs [7, 11–16]. These less favourable outcomes have been associated with bone quality and volume that are often more compromised in maxillary than in mandibular sites [11–16]; however, several factors such as the type, number, and position of the fixtures and the loading conditions may influence the treatment outcomes of implant-supported maxillary ODs [6, 7, 11–18]. A distinction has been introduced between “planned” and “unplanned” maxillary ODs, and better outcomes have been found for planned cases [8, 19]. A “planned” OD is the result of a sophisticated treatment planning, including an accurate preoperative radiographical assessment of the edentulous ridges, by means of cone beam computed tomography (CBCT) scan and three-dimensional (3D) reconstruction software, and the use of predefined operative criteria such as a minimum number of implants with sufficient length/diameter, inserted with the correct position/inclination and splinted with a rigid bar [8, 17–19]. In recent years, direct metal laser sintering (DMLS) has opened new frontiers in biomedical applications [20–23]. DMLS is a technology to directly generate physical objects on the basis of 3D computer models. This system uses precursor powders to build these shapes through a computer-controlled, self-assembly process. A high-power laser beam is directed on a metal powder bed and programmed to fuse particles according to a computer-controlled file, generating a thin metal layer; apposition and fusion of subsequent layers give shape to a desired 3D form, with minimal postprocessing requirements [20, 23–25]. In the last few years, the advancements in DMLS have allowed the fabrication of dental implants made of Ti and Ti-based alloys [23–25]. The chemical and mechanical properties of Ti dental implants fabricated with the DMLS technique have been investigated [20, 22–24]. Different* in vitro* studies with cultures of human osteoblasts [20, 25] and mesenchymal stem cells [26] have analyzed the biological response to DMLS implant surfaces, such as several* in vivo* histologic/histomorphometric studies in animals [21, 27–29] and humans [30–34]. Even though by now the concept of DMLS for implant manufacturing is well accepted, there is still limited clinical data in dental literature [35–41]; in addition, the only available prospective clinical studies are based on a small number of patient with a short follow-up [35–37].

The aim of the present 3-year prospective clinical study was to evaluate implant survival, complications, and peri-implant marginal bone loss of DMLS implants used to support bar-retained maxillary ODs.

## 2. Materials and Methods

### 2.1. Patient Selection

Between January 2009 and March 2011, all patients referred to the Dental Clinic of the University of Varese, Italy, and to a single private practice (Gravedona, Como, Italy) were considered for inclusion in this prospective clinical study.

Inclusion criteria were as follows:

- total edentulism in the maxilla, for a period of at least 3 months;
- functional problems with the conventional complete maxillary denture (lack of stability and/or discomfort);
- sufficient maxillary bone volume to place implants at least 3.3 mm in diameter and 8.0 mm in length.

Exclusion criteria were as follows:

- need for bone augmentation procedures with autogenous bone and/or bone substitutes prior to implant insertion;
- uncontrolled diabetes mellitus;
- immunocompromised status;
- radiotherapy in the maxillofacial region;
- chemotherapy;
- treatment with amino-bisphosphonates.

Smoking was not considered exclusion criteria for this study; however, patients were advised that smoking is associated with an increased risk of implant failure. All participants received detailed explanations about the planned treatment and its potential risks and complications and signed a written informed consent form prior to being enrolled in the study. The study was approved by the Local Ethical Committee, at the University of Varese, Italy, and was conducted in accordance with the principles outlined in the Declaration of Helsinki on Clinical Research Involving Human Subjects, 1975, as revised in 2002.

### 2.2. Implant Fabrication and Surface Characterization

**Figure 1:**
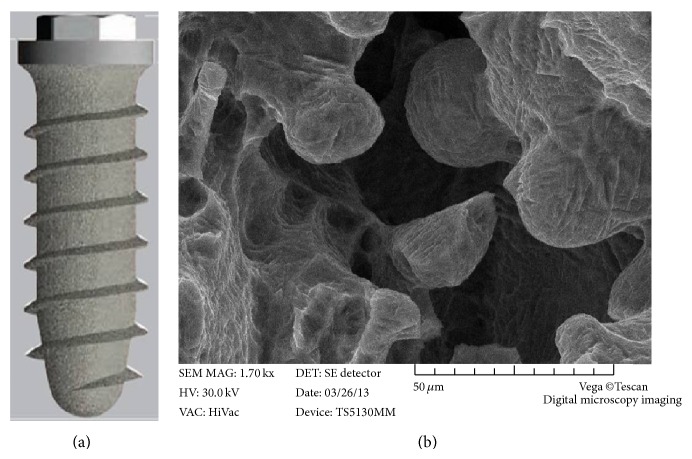
Macroscopical and microscopical features of the direct metal laser sintering (DMLS) titanium implants used in this study: the implants had an external hexagon connection (a); the scanning electron microscopy of the implant (×1700) showed a porous surface with ridge-like and globular protrusions, interspersed by intercommunicating pores and irregular crevices (b).

The implants were manufactured from titanium alloy (Ti-6Al-4V) with a DMLS technique (TixOs, Leader Implants, Milan, Italy). The DMLS implants were made of master alloy powder with a particle size of 25–45 micrometers as the basic material. Processing was carried out in an argon atmosphere using a powerful Yb (ytterbium) fiber laser system (Eosint 270, EOS GmbH, Munich, Germany) with the capacity to build a volume up to 250 × 250 × 215 mm using a wavelength of 1,054 nanometers with a continuous power of 200 W at a scanning rate of 7 m/s. The size of the laser spot was 0.1 mm. To remove residual particles from the manufacturing process, the sample was sonicated for 5 min in distilled water at 25°C, immersed in NaOH (20 g/L) and hydrogen peroxide (20 g/L) at 80°C for 30 min, and then further sonicated for 5 min in distilled water. Surface cleaning was completed by immersion of the samples in a mixture of 50% oxalic acid and 50% maleic acid at 80°C for 45 min, followed by washing for 5 min in distilled water in a sonic bath. The direct laser preparation provided an implant surface with a roughness that had an Ra value of 66.8, Rq value of 77.55, and Rz value of 358.3 micrometers, respectively. The implants for this study featured an external hexagon connection (Figure 1) and were available in lengths of 8.0, 10.0, 11.5, and 13 mm; the available diameters were 3.3, 3.75, and 4.5 mm.

### 2.3. Preoperative Workup

**Figure 2:**
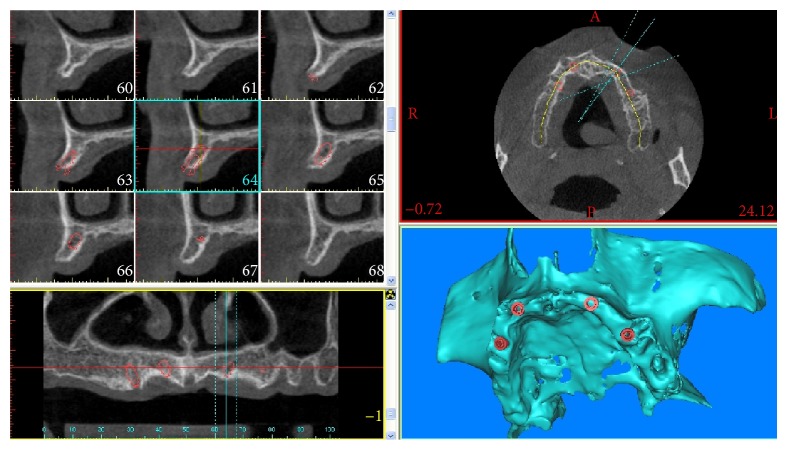
Three-dimensional (3D) reconstruction of the maxilla by means of implant navigation software, with virtual planning of implant placement.

A complete examination of the oral hard and soft tissues was carried out for each patient. Panoramic radiographs formed the basis for the primary investigation. Preoperative workups included an assessment of the edentulous ridges using casts and diagnostic wax-up. CBCT scans were used as final investigation. CBCT datasets were eventually transferred to specific implant navigation software (Mimics, Materialise, Leuven, Belgium) to perform a 3D reconstruction of the upper jaws. With this navigation software it was possible to correctly assess the width of each implant site and the thickness and the density of the cortical plates and the cancellous bone, as well as the ridge angulations (Figure 2).

### 2.4. Surgical and Prosthetic Procedures

**Figure 3:**
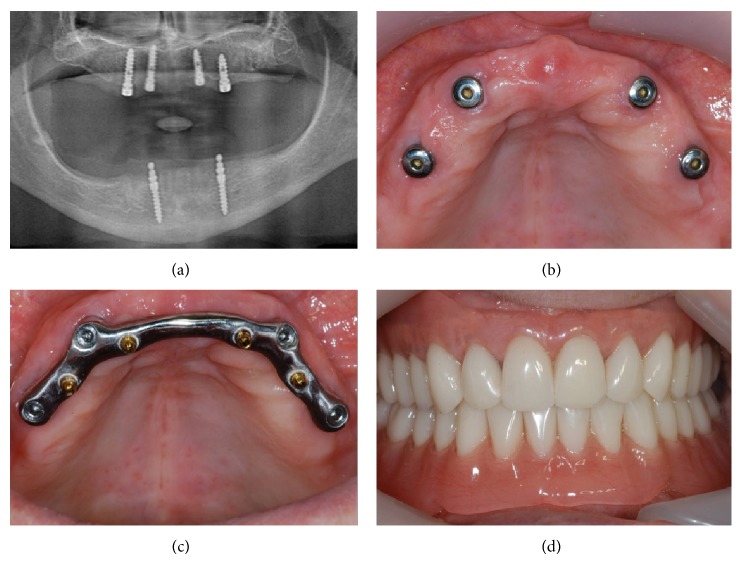
Surgical and prosthetic procedures: panoramic radiograph at implant placement (a); the implants after 4 months of undisturbed healing (b); the maxillary bar after application (c); clinical view of the maxillary overdenture (OD) at delivery (d).

The same experienced surgeon (C.M.) and prosthodontist (F.M.) performed all the surgical and prosthetic procedure, respectively, in the two clinical centers. Local anesthesia was obtained by infiltrating articaine 4% containing 1 : 100 adrenaline (Ubistesin, 3MEspe, St. Paul, MN, USA). An extended crestal incision was made, with or without releasing incisions, and full-thickness flaps were elevated exposing the alveolar ridge. The preparation of implant sites was carried out with spiral drills of increasing diameter (2.0 and 2.3 mm, to place an implant with 3.3 mm diameter; 2.0, 2.6, and 2.8 mm, to place an implant with 3.75 mm diameter; 2.0, 2.6, and 3.2 mm, to place an implant with 4.5 mm diameter) under constant irrigation. The fixtures were positioned at the bone crest level. Four implants were placed in each edentulous maxilla. The flaps were repositioned to cover the implants completely and were secured in position by interrupted sutures; after that, the patients' complete dentures were relined with a soft tissue conditioner (Soft-liner, GC Corporation, Tokyo, Japan) and used as temporary prostheses. Ice packs were provided postoperatively. All patients received oral antibiotics, amoxicillin + clavulanic acid 2 g each day for 6 days (Augmentin, Glaxosmithkline Beecham, Brentford, UK). Postoperative pain was controlled by administering 100 mg nimesulide (Aulin, Roche Pharmaceutical, Basel, Switzerland) every 12 h for 2 days, and detailed instructions about oral hygiene were given, with mouth rinses with 0.12% chlorhexidine (chlorhexidine, Oral B, Boston, MA, USA) administered for 7 days. Patients were instructed to eat a soft diet for 7 days. Smokers were told to avoid smoking for 48 hours postoperatively. Suture removal was performed at 8–10 days. Again, the provisional complete dentures were relined with a soft tissue conditioner. All patients wore the provisional complete dentures before returning for second-stage surgery. The implants were left submerged for 4 months. After this undisturbed healing period, second-stage surgery was conducted to gain access to the underlying implants and healing abutments were placed. A mesiodistal crestal incision, limited to the implant sites, was made and the ridge mucosa was elevated to uncover the implants; then, cover screws were replaced by healing abutments. The mucosal flap was adjusted to the healing abutment and sutured in position. After that, the complete provisional dentures were discharged abundantly around the healing abutments, seated in mouth, and another partial relining with a tissue conditioner was performed. Two weeks later, the healing abutments were removed and pick-up impression posts were placed at the implant level. The final implant impression was made with generic trays using polyvinylsiloxane (Aquasil Monophase, Dentsply International, York, PA, USA). From this impression, a master cast was poured, and a bar was fabricated. For all patients, the splinting suprastructures for the implants consisted of a rigid cobalt-chrome bar, without extensions. Each bar was supported by four implants and presented four boxes for insertion of precision attachments. After the fabrication of the bar, the implants were elongated with prefabricated titanium abutments, to the top of which titanium copings were screwed. All ODs had a horseshoe design and were fabricated with acrylic resin with a metal framework. Retention of the superstructure was obtained from four prefabricated precision attachments, consisting of nitrided titanium balls (Pivots, Rhein 83 srl, Bologna, Italy) cemented in the boxes of the bar with zinc-phosphate cement (Harvard, Richter & Hoffmann GmbH, Hoppegarten, Germany) (Figure 3). The same dental technician fabricated the bars and the ODs. All ODs were carefully evaluated for proper occlusion: protrusion and laterotrusion were assessed on the articulator and intraorally, to secure a balanced occlusion in centric relation without anterior tooth contact.

### 2.5. Outcome Measures

After the delivery of the OD, all patients were included in a maintenance program, which comprised professional oral hygiene every 6 months. During each follow-up visit, a clinical assessment of implants, peri-implant tissues, and prostheses was conducted by a periodontist and a prosthodontist neither of whom were directly involved in the treatment of these patients. The following outcome measures were evaluated:

- implant survival. Implant losses were all categorised as failures. The conditions for which implant removal could be indicated included implant mobility due to failure of osseointegration, peri-implant infection with pain/suppuration, progressive marginal bone loss due to mechanical overload, and implant body fracture. A distinction was made between “early” (before the abutment connection) or “late” (after the abutment connection) implant failures;
- biological complications, including pain or swelling after surgery, soft tissue inflammation, and peri-implant infection with fistula formation, pain, suppuration, or exudation. The threshold of peri-implantitis was indicated by a probing pocket depth ≥6 mm and bleeding on probing or pus secretion;
- prosthetic complications, such as mechanical complications related to implant components (loosening or fracture of abutment), and technical complications including issues related to anchorage structure (broken bars, or loose, lost, or broken attachments) or prostheses (repairs of fractured prostheses or overdenture teeth). Static and dynamic occlusions were evaluated using standard occluding papers (Bausch articulating paper, Bausch inc, Nashua, NH, USA);
- peri-implant marginal bone loss. Intraoral periapical radiographs were taken of each implant, using a Rinn alignment system (Rinn, Dentsply, Elgin, IL, USA) with a rigid film-object-X-ray source coupled to a beam-aiming device in order to achieve reproducible exposure geometry. Radiographs were taken at the baseline (immediately after implant insertion) and at each annual follow-up session: customised positioners were used to help the correct repositioning of the radiographic template. Changes in peri-implant marginal bone level, as modifications in the distance from the implant shoulder to the first visible bone-to-implant contact (DIB), were measured on periapical radiographs which were taken immediately after installation and at the 1-, 2-, and 3-year follow-up examinations. The DIB was measured in mm, at the mesial and distal implant side of each implant, with the aid of an ocular grid. In order to correct for dimensional distortion, the apparent dimension of each implant was measured on the radiograph and then compared with the real implant length; mean values between the mesial and the distal measures were obtained for each implant.

### 2.6. Statistical Analysis

Data analysis was performed by an independent examiner who was not directly involved in the study. Databases were created with worksheet (Microsoft Excel, Microsoft Corporation, Redmond, WA, USA) and used for the analysis. Descriptive statistics were used for patient demographics (gender, age, and smoking habit) and distribution of implants (position, diameter, and length). Absolute and relative frequency distributions were calculated for qualitative variables, such as implant survival/failure and the incidence of biological and prosthetic complications; means, standard deviations, medians, and confidence intervals (95%) were calculated for quantitative variables, such as peri-implant bone resorption. Implant survival, complications, and peri-implant bone resorption were calculated at the implant and at the patient level.

## 3. Results

### 3.1. Patient Population and Implant-Supported ODs

**Figure 4:**
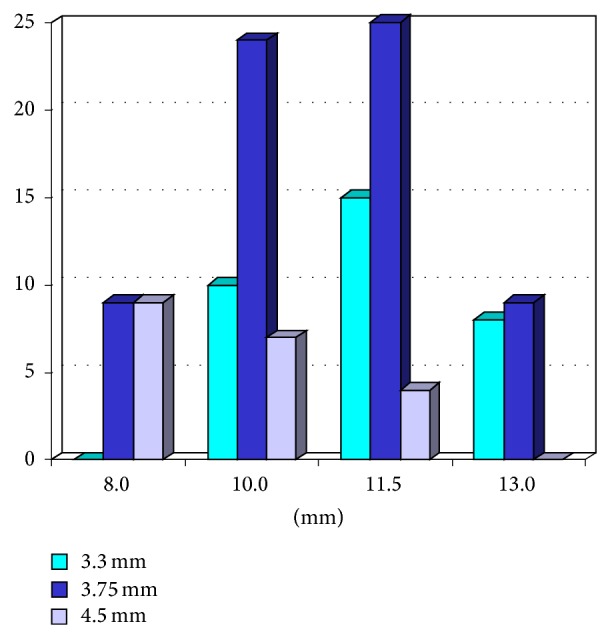
Distribution of the implants by length and diameter.

In total, 35 patients (20 males and 15 females) were considered for inclusion in the present study, over a 2-year period (January 2009–March 2011), in two different clinical centers. However, 5 patients presented conditions enlisted in the exclusion criteria (one for immunocompromised status, two for uncontrolled diabetes mellitus, and two for treatment with amino-bisphosphonates) and could not be included in the study. Consequently, with regard to the aforementioned inclusion and exclusion criteria, only 30 patients (18 males and 12 females; mean age 70 ± 5.5 years, range 62–81, median 69, CI 95%: 68.1–71.9) were enrolled in the present study. Among these patients, 12 (40%) were smokers. All patients had conventional complete dentures or implant-supported ODs in the mandible. In total, 120 implants were placed: 60 in the maxillary lateral incisor and 60 in the first premolar area. The distribution of implants by length and diameter was in accordance with Figure 4. Thirty maxillary ODs, each one supported by 4 implants, were delivered.

### 3.2. Implant Survival

**Figure 5:**
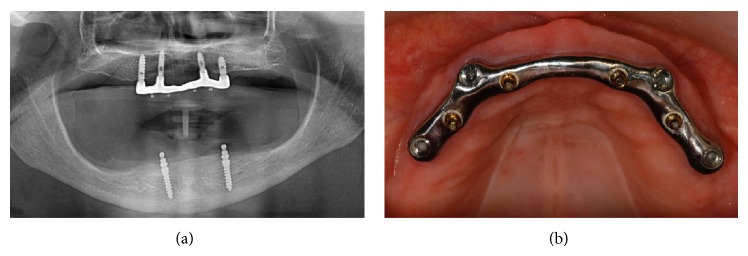
The maxillary overdenture (OD) at the 3-year follow-up: panoramic control radiograph (a); clinical view of the bar (b).

Only 28 patients (112 implants) were available for the 3-year follow-up control. In fact, 2 patients (8 implants) could not attend the final follow-up examination (one patient died; another had serious health problems, not related to the dental implant therapy, and was hospitalized) and were considered as dropouts. At the end of the study, the overall 3-year implant survival rate was 97.4% (implant-based) and 92.9% (patient-based) with 109 implants still in function (Figure 5). Three implants failed and had to be removed. Two fixtures failed in a 68-year-old male smoking patient. These implants were classified as “early failures” showing clinical mobility due to a lack of osseointegration, without clinical signs of peri-implant infection; all these failures occurred 4 months after placement, in the first premolar areas, before the connection of the abutment. Another implant failed for extensive bone loss due to recurrent peri-implantitis with pain and suppuration, in a 72-year-old smoking female patient. This failure occurred in the premolar area after 1 year of function and was consequently classified as “late failure.”

### 3.3. Biological Complications

One 69-year-old nonsmoking female patient (two implants) experienced pain and swelling after surgery. However, the pain was managed by giving anti-inflammatory and analgesic medication, and no further complications were reported for this patient. Two years after placement, one 70-year-old smoking male patient was diagnosed with peri-implantitis: in fact, two implants (one lateral incisor and one first premolar) showed suppuration/exudation, bleeding on probing and a probing depth ≥6 mm. However, this patient was successfully treated, and no further biological complications were recorded. At the end of the study, the incidence of biological complications was 3.5% (implant-based) and 7.1% (patient-based).

### 3.4. Prosthetic Complications

No mechanical complications related to implant components (abutment loosening, abutment fractures) were reported. The anchorage components used for connecting the bar to the prosthetic framework showed no complications. All the prosthetic complications were technical in nature and consisted in repairs of fractured prostheses (acrylic resin was recorded in 2 patients) or OD teeth (tooth fracture occurred in 3 patients). At the end of the study, the incidence of prosthetic complications was 17.8% (patient-based).

### 3.5. Peri-Implant Marginal Bone Loss

**Figure 6:**
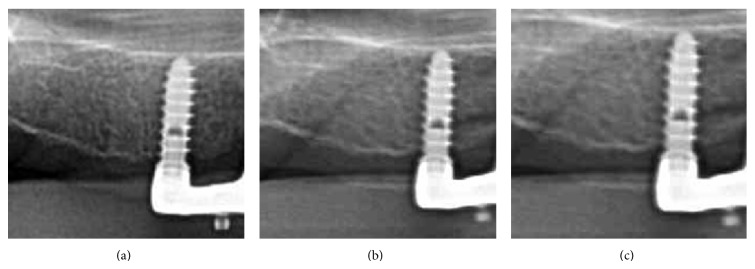
Periapical radiographs of an implant at the 1-year (a), 2-year (b), and 3-year (c) follow-up examination, respectively.

The overall radiographic evaluation of the implants showed a mean distance from the implant shoulder to the first visible bone-to-implant contact (DIB) of 0.39 mm (±0.25, median 0.4, CI 95%: 0.35–0.43) at the 1-year examination. At the 2-year follow-up control, the bone level of the fixture was situated 0.5 mm (±0.23, median 0.55, CI 95% 0.46–0.54) from the reference point. Finally, at the 3-year examination, the bone level of the fixture was situated 0.62 mm (±0.28, median 0.6, CI 95% 0.57–0.67) from the reference point. No detrimental effects on marginal bone level were evident between the 1- and 3-year follow-up examinations (Figure 6).

## 4. Discussion

In contrast to the excellent long-term implant survival and success rates shown for implant-supported mandibular ODs [4–6], considerably lower survival and success rates have been reported in several studies on maxillary ODs [7, 11–16]. In a retrospective multicenter evaluation of osseointegrated implants supporting ODs, Engquist et al. [11] showed a significantly higher loss of fixtures in the maxilla then in the mandible. In another retrospective study on maxillary ODs retained by splinted and unsplinted implants, Närhi et al. [12] reported a cumulative survival rate for the implants after 72 months of 90%. In a retrospective 10-year follow-up study, Schwartz-Arad et al. [13] reported more complications and implant failures with implant-supported ODs in the maxilla (83.0% survival) than in the mandible (99.5% survival). Similar results were described by Visser et al. [7] in a 10-year follow-up study on maxillary ODs supported by six endosseous implants and a milled bar mesostructure, with an overall implant survival rate of 86.1%. More recently, in a 3-year prospective clinical study on 95 patients with 107 ODs supported by 360 implants, Balaguer et al. [16] found a significantly lower implant survival in the maxilla (91.9%) than in the mandible (98.6%). All these results have been related to poor bone quantity/quality or short implants inserted in severely resorbed maxillae [7, 11, 13, 16]. Poor bone quality, low bone quantity, short implant length with reduced diameter, and poor initial stability are potential problems encountered in the edentulous maxilla and may be responsible for a higher risk of implant loss with maxillary ODs [7, 11–16]. More recently, however, several studies have suggested that “planned” implant placement for maxillary OD treatment may have a better outcome than emergency procedures, in which the placement of an insufficient number of fixtures and/or previous failures made a fixed full dental prosthesis an unfeasible option [8–10, 14, 19, 42]. In a 5-year follow-up retrospective evaluation based on twenty-seven subjects, of whom 13 were originally planned for OD treatment and the other 14 for a fixed prosthesis, Widbom et al. [14] found that fewer implant failures occurred in patients originally planned for OD treatment. In a study on “planned” implant-supported maxillary ODs, Krennmair et al. [42] have shown a cumulative 5-year survival rate higher than 98% with four implants placed in the maxillary anterior region anchored on a milled bar. In this work, the authors have also demonstrated that implant placement in the posterior maxillary region for OD anchoring may guarantee an excellent survival rate, even after sinus augmentation [42]. In another “planned” study, Sanna et al. [19] showed a good outcome with four to six interconnected implants supporting an OD in the maxilla, with a cumulative survival rate of the supporting implants of 99.3% after 10 years of function. These results were confirmed by a recent 5-year prospective clinical study on “planned” bar-retained ODs supported by locking-taper implants [8], in which satisfactory survival and success rate were achieved both in the mandible (98.6%) and in the maxilla (97.4%). These studies demonstrate that the use of a minimum number of four splinted implants of sufficient length and diameter, as well as a careful preoperative planning of implant placement with an accurate study of bone quality and quantity, can result in high survival and success rates of maxillary implants supporting ODs [8, 14, 19, 42], as confirmed by several reviews [18, 19]. Our present 3-year follow-up study on DMLS titanium implants supporting bar-retained maxillary ODs seems to confirm the results of previous studies on “planned” ODs, with excellent outcomes in terms of implant survival (97.4% implant-based; 92.9% patient-based) and a low incidence of biological complications (3.5% implant-based; 7.1% patient-based). Only three implants failed, in two smoking patients. Smoking is a well-documented risk factor for implant failure [43], and the results of our study seem to confirm this evidence. Some prosthetic complications (17.8%) were encountered during this study; however, all these complications were technical in nature and consisted in acrylic resin or OD teeth fractures. Finally, at the 3-year examination, a mean DIB of 0.62 mm (±0.28) was found; minimal changes were observed in the bone level between the 1- and 3-year follow-up examinations, thus in accordance with other recent studies [17, 18, 44, 45]. DMLS offers the possibility to fabricate titanium dental implants with a bulk, high-density core and a porous, low-density surface, thus combining a solid material with a porous material (with different percentages of porosity) in one single structure [20–24]. The main advantage of the DMLS, in fact, is the possibility to fabricate implants with a precisely controlled porous surface [20, 23, 25]. This layered manufacturing technique provide a complete control over both percentage porosity and the geometry of the interconnected open pore network, so that an intricate, highly porous surface can be prepared directly from metal powders, with minimal postprocessing requirements [23, 25]. This highly porous surface provides ample space for bone regeneration, since bone tissue can grow into the pores to integrate with them: this may improve long-term implant fixation [23, 25, 27–34]. In addition, DMLS enables the fabrication of “functionally graded” implants, with a gradient of porosity perpendicular to the long axis. With this approach, the mechanical properties of the titanium implant can be tailored to better match the yield strength and elastic modulus of the host bone, therefore reducing the “stress-shielding” effect. This approach introduces the concept of “isoelastic” dental implants, even if the fatigue properties of highly porous biomaterials may suffer from high levels of porosity [20, 23, 25, 27–34]. Finally, another important advantage of DMLS is to have the unlimited freedom and ability to fabricate highly complex structures: with this layered manufacturing technique, “custom-made” patient-specific implants can be produced in a cost-time competitive manner [38–41].

## 5. Conclusions

Based on these results, and within the limits of this study (such as the limited number of patients treated and fixtures placed, absence of a control group, and absence of calibration of the evaluators), the use of four DMLS titanium implants to support bar-retained maxillary ODs seems to represent a safe and successful procedure, with excellent 3-year survival rates (97.4% implant-based; 92.9% patient-based) and a low incidence of biological (3.5% implant-based; 7.1% patient-based) complications. Some prosthetic complications (17.8%) were reported. No detrimental effects on marginal bone level were evident at the end of the study, with a 3-year mean DIB of 0.62 ± 0.28 mm. Further, long-term clinical studies on a larger sample of patients are needed to confirm these results.
